# Diagnostic accuracy of linked colour imaging versus white light imaging for early gastric cancers: a prospective, multicentre, randomized controlled trial study

**DOI:** 10.1080/07853890.2022.2147991

**Published:** 2022-11-21

**Authors:** Min Min, Xiaotian Sun, Jianying Bai, Qinsheng Zhang, Xiaocui Yang, Qiang Guo, Rong Wang, Bangmao Wang, Zhiwu Lv, Jie Pan, Chunmeng Jiang, Duanmin Hu, Bing Nong, Enqiang Linghu, Yan Liu

**Affiliations:** a Department of Gastroenterology, The Fifth Medical Center of Chinese PLA General Hospital, Beijing, China; b Department of Gastroenterology, Xinqiao Hospital, Third Military Medical University, Chongqing, China; c Hepatobiliary & Gastrointestinal Ward 3, Henan Province Hospital of TCM, Zhengzhou, China; d Department of Gastroenterology, Ankang City Center Hospital, Ankang, China; e Department of Gastroenterology, The First People’s Hospital of Yunnan Province, Kunming, China; f Digestive Endoscopy Center, Shanxi Provincial people’s Hospital, Taiyuan, China; g Department of Gastroenterology, Tianjin Medical University General Hospital, Tianjin, China; h Department of Gastroenterology, People’s Hospital of Baoan District, Shenzhen, China; i Department of Gastroenterology, Wenzhou Central Hospital, Wenzhou, China; j Department of Gastroenterology, The Second Hospital of Dalian Medical University, Dalian, China; k Department of Gastroenterology, The Second Affiliated Hospital of Soochow University, Suzhou, China; l Department of Digestion, The People’s Hospital of Guangxi Zhuang Autonomous Region, Nanning, China; m Department of Gastroenterology, The First Medical Center of Chinese PLA General Hospital, Beijing, China

**Keywords:** Linked colour imaging, white light imaging, early gastric cancer, randomized controlled trial, diagnostic accuracy

## Abstract

**Background:**

Linked colour imaging (LCI) is a novel new image-enhanced endoscopy (IEE) technology that produces bright and vivid images. The aim of this study was to assess the ability of LCI to improve the diagnostic accuracy of early gastric cancer (EGC) relative to white light imaging (WLI).

**Materials and methods:**

We performed this study on patients undergoing screening endoscopy from 12 medical institutions in China. Patients were randomly assigned to receive WLI followed by LCI or LCI followed by WLI. The primary outcome was to compared the diagnostic accuracy between LCI and WLI for EGC/high-grade intraepithelial neoplasms. Secondary outcomes included the numbers of suspicious lesions, neoplastic lesions and examination time by using LCI detected versus using WLI.

**Results:**

A total of 1924 patients were randomly selected, and 1828 were included in the analysis. The diagnostic accuracy for EGC, which was 78.8% by using LCI and 68.4% by using WLI (*p* < .0001). More suspicious lesions were detected by LCI than by WLI (*n* = 1235 vs. 1036, *p* = .031), especially among differentiated EGC (*p* = .013). LCI greatly shortened the examination time compared with WLI (*p* = .019).

**Conclusions:**

LCI has better accuracy and shorter examination time in diagnosing EGC than WLI (Clinical trial registration: NCT03092414).

**Key messages**
- Compared with white light imaging (WLI), the diagnostic accuracy, sensitivity and specificity increased by using LCI.
- More lesions were detected by LCI alone than by WLI alone, especially among differentiated EGC.
- LCI may be used as a screening tool for routine clinical observation.

## Introduction

Gastric cancer is the seventh leading cause of death in China (1). The detection of gastric cancer at an early stage correlates with a good prognosis. However, the diagnosis of early gastric cancer (EGC) using conventional white light imaging (WLI) is occasionally difficult and has unsatisfactory sensitivity (2,3)_._ Although new image-enhanced endoscopy (IEE) techniques, such as narrow band imaging (NBI) and blue laser imaging (BLI), are more useful than conventional WLI in detecting and diagnosing EGC, their effectiveness is still criticized because they may not provide sufficient brightness for a thorough examination of organs with large luminal diameters, such as the stomach, making them inappropriate for screening endoscopy (4,5). Furthermore, the efficacy of IEE without magnification in the detection of EGC remains controversial. To date, valid screening procedures for EGC have been lacking, and perfect endoscopic methods for the detection of EGC remain difficult to implement in current clinical practice; there is an urgent need to resolve these issues.

Linked colour imaging (LCI; Fujifilm Co., Tokyo, Japan) is a novel IEE technology that produces bright and vivid images for distant views and provides a brighter view of the gastric lumen than WLI can provide (6–8). Several studies have demonstrated LCI to be more effective than WLI in the detection of gastrointestinal lesions (9–11). LCI enables superior endoscopic detection of superficial lesions throughout the gastrointestinal tract by enhancing the colour contrast between the neoplasm and the surrounding mucosa. Therefore, LCI is the more suitable of the two for detecting flat lesions of the gastric mucosa, which is very difficult with WLI (12).

LCI enhances slight colour differences and theoretically enhances the identification of suspicious lesions. Failure to carry out selective biopsy or magnifying endoscopy for suspicious lesions seriously affects the diagnostic efficiency, however. Previous studies have demonstrated that targeting biopsies according to the type of colour change could yield potential benefits (13,14), delineating EGCs (described as orange–red (7) or red with yellow in the centre (13)) surrounded by intestinal metaplasia (purple in colour). Therefore, the acquisition of LCI-based endoscopic images to predict EGC may improve the diagnostic efficiency of screening endoscopy. Based on the above, LCI is expected to facilitate new diagnostic approaches to gastric lesions and may be an effective screening method for detecting EGCs.

A prospective, multicentre randomized controlled trial (RCT) should be performed to investigate the true efficacy of LCI in diagnosing EGC through endoscopy and determine whether LCI can be used as a screening tool during routine clinical examinations. Our study aims to compare the diagnostic accuracy of LCI to that of WLI for EGC on screening endoscopy and to assess the effectiveness of LCI in recognizing EGC.

## Methods

This prospective, multicentre RCT was conducted at 12 medical institutions. The study period was from May 2017 to March 2018. According to the number of routine endoscopy cases, six relatively small institutions were expected to enroll at most 100 patients each, while six larger institutions were expected to provide more than 200 patients each (ratio 1:1). This study was approved by the institutional review board of the Fifth Medical Center of Chinese PLA General Hospital and was performed in accordance with the Declaration of Helsinki. Written informed consent was obtained from all patients, and the study was registered with Clinical Trials (NCT03092414).

## Patients

Consecutive patients who underwent upper gastrointestinal (GI) endoscopy were enrolled in this study. The inclusion criteria were age >18 years, indications for upper GI endoscopy and consent to participate in the study. The exclusion criteria were as follows: known advanced cancer; previous stomach surgery; or unsuitability conditions making the patient ineligible for biopsy, such as nonsteroidal anti-inflammatory drug administration, severe uncontrolled coagulopathy, impaired renal function, liver cirrhosis, pregnancy, lactation, an inability to provide informed consent or a reluctance to participate in this study.

## Randomization and masking

**Figure 1.**
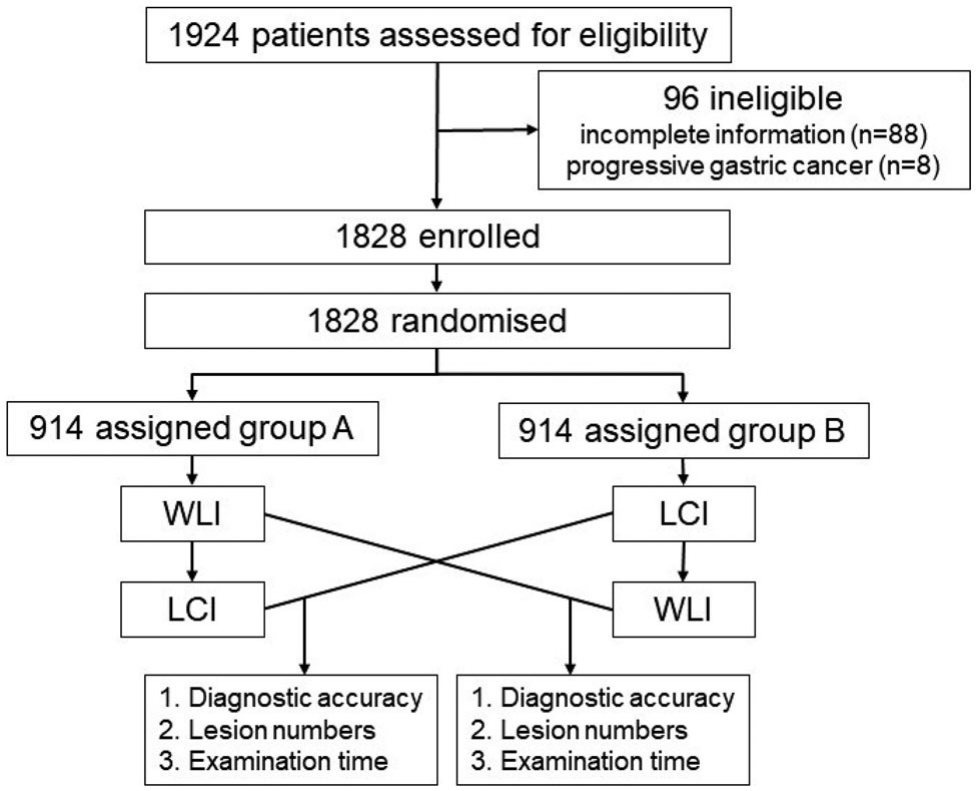
Flow chart of this study.

Before upper GI endoscopy was initiated, patients were randomized in a 1:1 ratio to the WLI-LCI group (WLI followed by secondary LCI) or the LCI-WLI group (LCI followed by secondary WLI). The randomization was based on a computer-generated randomized block sequence (block = 4). A study flowchart is shown in Figure 1. The procedures were performed by 28 endoscopists (2 or 3 endoscopists at each institution) who had each performed at least 150 LCI examinations before the start of the study. At all locations, the duration of each stomach examination was extracted from the video files from start to finish for each location. The endoscopes used were EG-L590WR units with LASEREO (Fujifilm Co, Tokyo, Japan).

To standardize the endoscopic findings, all participating endoscopists completed an one-day formal training session on the characteristics of suspicious lesions on LCI and WLI before study initiation, with the goal of familiarizing them with the diagnostic criteria and making their diagnostic accuracy uniform, particularly when in the use of LCI. During training, the endoscopists were shown a PowerPoint presentation comprising representative LCI and WLI images of EGCs and the EGC diagnostic criteria with expert commentary in a face to face session; these images were used to interpret the endoscopic characterization of the EGCs. The training module was structured as an overview of the contents of diagnostic criteria under LCI. In both groups, the gastric lumen was observed, biopsy specimens were taken from suspicious lesions, and a pathological diagnosis was made.

## Definition of endoscopic findings and procedures

**Figure 2.**
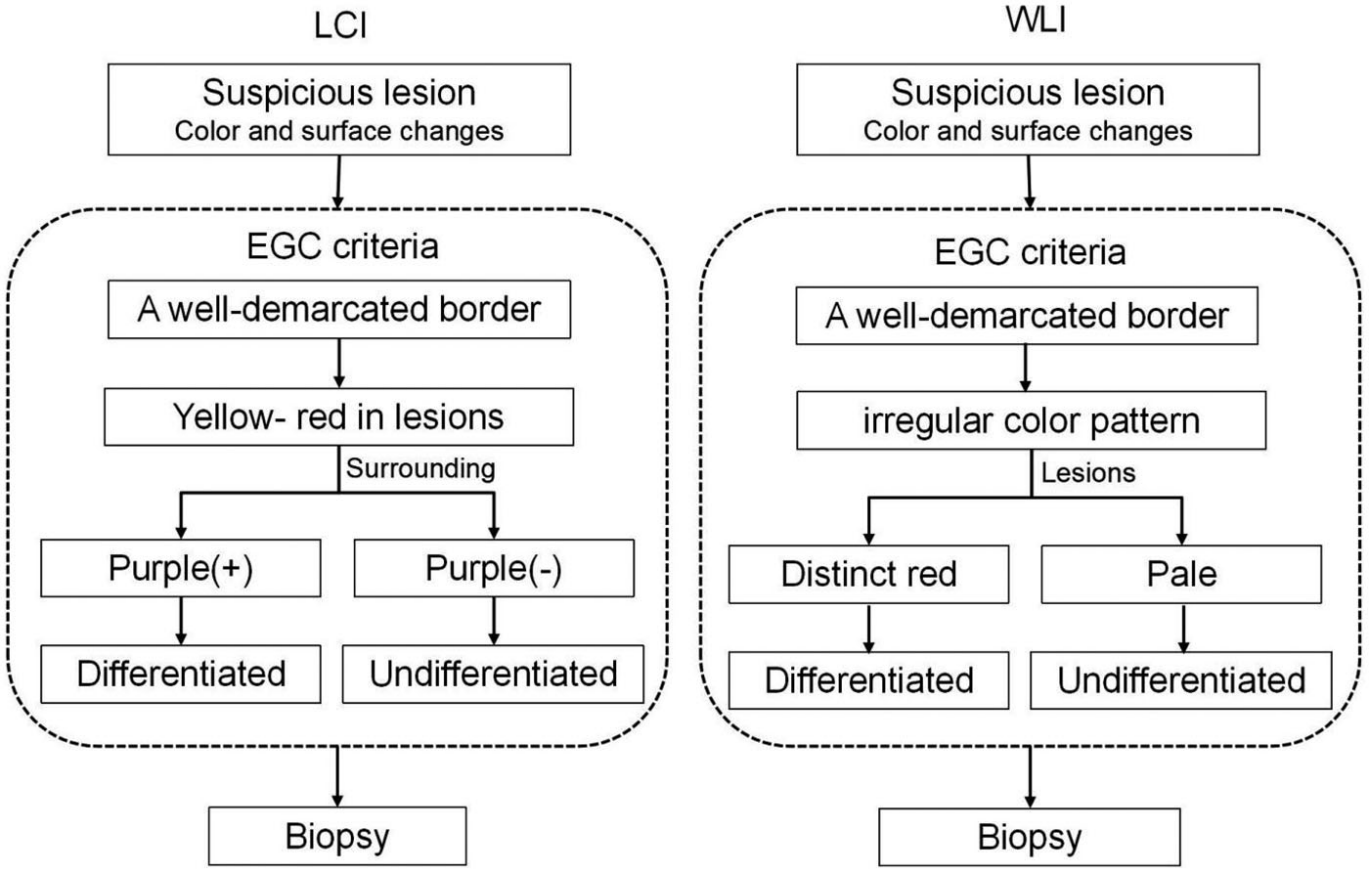
Linked colour imaging (LCI) and white light imaging (WLI) diagnostic diagram for early gastric cancer (EGC). The diagnosis of EGC under WLI was defined as a presence of a well-demarcated border and an irregular colour/surface pattern, then according to distinct red or pale in colour of the lesion was categorized into differentiated or undifferentiated type. The diagnosis of EGC under LCI was defined as a presence of a well-demarcated border and a yellow-red colour in the suspicious lesion, then according to the presence of purple in the peripheral mucosa, the lesion was categorized into differentiated or undifferentiated type.

**Figure 3.**
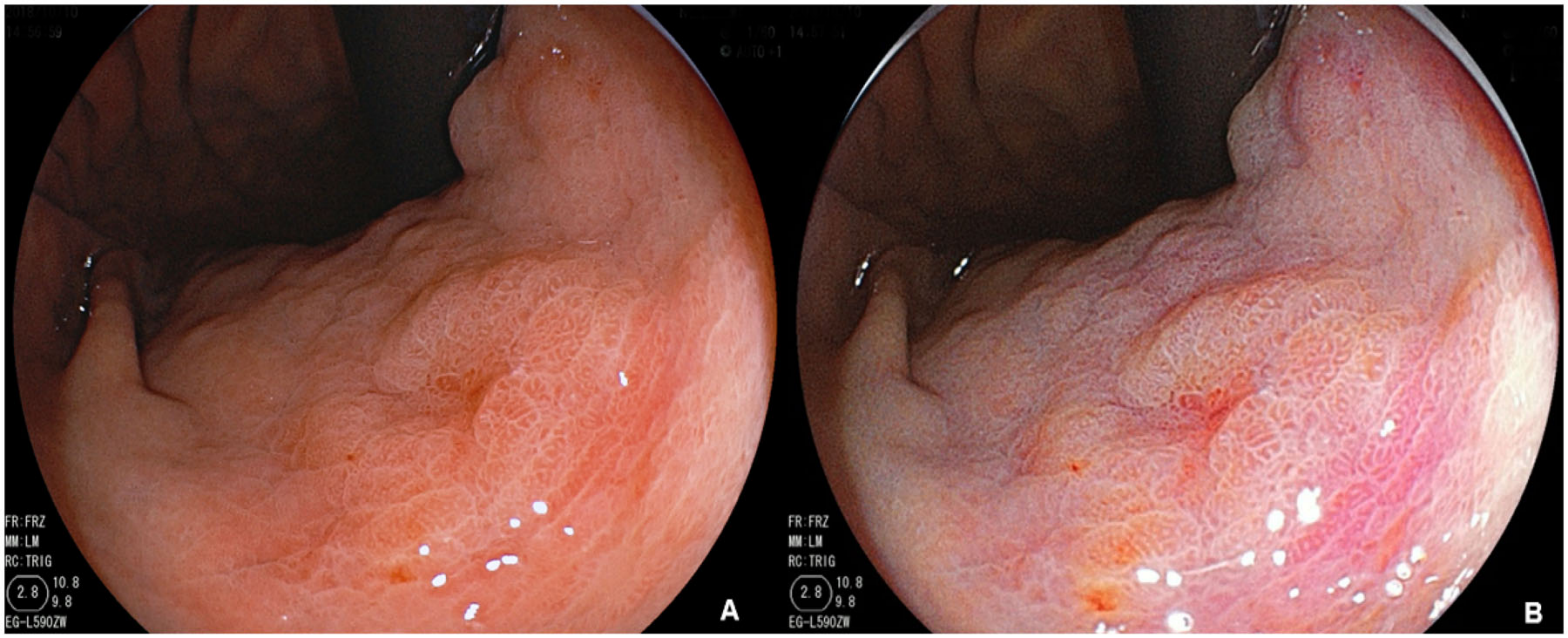
Representative endoscopic images of early gastric cancer (EGC) by using WLI(A) and LCI(B).

Because endoscopists generally detect EGCs based on the suspicious lesions, the definition of suspicious lesions was standardized in this study, the suspicious lesion was characterized as the presence of colour and surface changes of the mucosa (15). A diagnosis of EGC by WLI observation was defined in a previous study (16), then distinct red and pale in colour of the lesions were categorized into as differentiated or undifferentiated type. Besides the presence of a well-demarcated border, based on our previous study, a yellow-red colour in the suspicious lesion was included to in the definition of EGC by using LCI, and lesions were classified as differentiated or undifferentiated according to the presence of purple in the peripheral mucosa, the lesion was categorized into differentiated or undifferentiated type. The diagnostic diagram and typical endoscopic images are shown in Figures 2 and 3. First, whether there is a well-demarcated border to distinguish between cancer and non-cancer lesions, second, if the lesion has the colour which yellow-red (EGC) in the centre and surrounding by purple (intestinal metaplasia), it defined as differentiated EGC, which not surrounding by purple was defined as undifferentiated EGC.

Lesion specimens were obtained and fixed with 10% formalin. EGC was defined as gastric cancer occurring in the gastric mucosa and confined to the mucosa or submucosa irrespective of lymph node metastasis (17). Neoplastic lesions were defined as high-grade intraepithelial neoplasms (HGINs) or carcinomas in the stomach. Histopathological diagnosis was performed according to the revised Vienna classification.

## Outcome measures

The primary outcome of this study was the diagnostic accuracy of LCI and WLI for EGC/HGINs during screening. The secondary outcomes were the number of suspicious lesions, the number of neoplastic lesions and the examination time with the use of LCI versus WLI. The histopathological diagnoses of the obtained biopsy specimens were used as the gold standard for the diagnosis.

## Sample size calculation and statistical analysis

The sensitivity and specificity of WLI for EGC are 40% and 68%, respectively, according to the literature (4). On the basis of our preliminary experiment, we assumed that LCI had a sensitivity ≥0.60 and a specificity ≥0.70. Using a two-sided test with a 95% confidence interval and an alpha of 0.05, the required number of suspicious lesions was 691. Next, the required number of patients was calculated with reference to the suspicious lesion detection rate in endoscopic screening (the lower bound was approximately 50% based on prior data from the participating medical institutions); with an assumed dropout rate of 10%, the minimum number of enrolled patients was 1520.

Accuracy, sensitivity, specificity, positive predictive value (PPV), negative predictive value (NPV) and 95% confidence intervals (CIs) were calculated for those features showing significant differences. Continuous variables are expressed as the mean and range (SD) or median and interquartile range. Fisher’s exact test was used to analyse categorical data and compare proportions, and the *t* test was used to analyse continuous data. McNemar’s test was used to compare the diagnostic sensitivity, specificity and accuracy of LCI and WLI for EGC. SPSS software version 26 (SPSS Inc., Chicago, IL, USA) was used to conduct all analyses. All probability values calculated in this analysis were two-sided, and *p* < .05 was considered significant.

## Results

### Patient characteristics

A total of 1924 patients undergoing routine esophagogastroduodenoscopy (EGD) examinations were enrolled. After the exclusion of 96 patients (88 patients who had incomplete information, as well as 8 patients who had progressive gastric cancer), the study cohort consisted of 1828 patients (1038 males and 790 females). In these 1828 patients, diagnosis was the main indication for undergoing EGD; 195 patients were undergoing screening, and 51 patients were undergoing surveillance. All patients were randomly divided into two groups, namely, the WLI-LCI group (*n* = 914) and the LCI-WLI group (*n* = 914), using block randomization. The characteristics of the patients are summarized in Table 1. The two groups were comparable with respect to demographic features. The baseline characteristics of the two groups were well balanced. As shown in Table 1, there were no statistically significant differences in age, sex, family history or *Helicobacter pylori* (Hp) infection. Among the patients, 57 gastric EGCs/HGINs were detected, 44 of which were differentiated adenocarcinomas/HGINs and 13 of which were undifferentiated adenocarcinomas (Table 1).

**Table 1.** Demographic and clinical characteristics.

	WLI-LCI(*n* = 914)	LCI-WLI (*n* = 914)	*p* Value
Age, years			
Median (range)	54 (18–67)	59 (18–69)	.130
Gender, *n* (%)			.220
Female	408 (44.6)	382 (41.8)	
Male	506 (55.4)	532 (58.2)	
Family history of gastric cancer, *n* (%)	202 (22.1)	231 (25.3)	.111
No *H. pylori* infection	254 (27.8)	305 (33.4)	
*H. pylori* eradication	297 (32.5)	286 (31.3)	
Non-*H. pylori* eradication	363 (39.7)	323 (35.3)	
Indication			
Screening	99 (10.8)	96 (10.5)	.820
Surveillance	27 (3.0)	24 (2.6)	.670
Diagnosis	788 (86.2)	794 (83.9)	.681
Symptoms, *n* (%)			
Abdominal distension	812 (88.8)	798 (87.3)	.312
Abdominal pain	651 (71.2)	648 (70.9)	.877
Dyspepsia	677 (74.1)	645 (70.6)	.094
Heartburn	433 (47.4)	456 (49.9)	.282
Suspicious lesion			
LCI mode	642	593	.242
WLI mode	575	461	.652
Pathological diagnosis, *n* (%)			
Cancer/HGINs	27 (3.0)	30 (3.3)	.686
Differentiated adenocarcinoma/HGINs	22 (2.4)	22 (2.4)	1.000
Undifferentiated adenocarcinoma	5 (0.5)	8 (0.9)	.404

### Diagnostic accuracy, sensitivity and specificity

The primary outcome of our study was the diagnostic accuracy for EGCs/HGINs, which was 78.8% (95% CI, 76.4%–81.0%) with LCI and 68.4% (95% CI, 65.5%–71.2%) with WLI (Table 2). The diagnostic accuracy of LCI was approximately 10% higher than that of WLI (*p* < .0001). LCI also had greater sensitivity than WLI in diagnosing EGCs/HGINs (89.5% (95% CI, 78.9%–95.1%) vs. 59.7% (95% CI, 46.7%–71.4%); *p* = .0003). The diagnostic specificity values of LCI and WLI were 78.3% (95% CI, 75.9%–80.5%) and 69.0% (95% CI, 66.0%–71.8%) (*p* < .0001), respectively. The PPVs for EGCs/HGINs were 16.6% and 10.1% with LCI and WLI, respectively (*p* = .014). The NPVs for EGCs/HGINs were 99.3% and 96.7% with LCI and WLI, respectively (*p* < .0001).

**Table 2.** Diagnostic efficacy evaluation of WLI and LCI for EGC/HGINs.

	Accuracy (95% CI)	Sensitivity (95% CI)	Specificity (95% CI)	PPV (95% CI)	NPV (95% CI)
LCI	0.7879 (0.7642, 0.8097)	0.8947 (0.7888, 0.9509)	0.7827 (0.7582, 0.8053)	0.1661 (0.1287, 0.2118)	0.9935 (0.9860, 0.9970)
WL	0.6844 (0.6554, 0.7119)	0.5965 (0.4670, 0.7138)	0.6895 (0.6598, 0.7177)	0.1006 (0.0729, 0.1373)	0.9670 (0.9510, 0.9779)
*p* Value	<.0001	.0003	<.0001	.0140	<.0001

### Additional results

Table 3 indicates the endoscopic findings and durations of the EGD examinations by LCI and WLI. More suspicious lesions were detected by LCI than by WLI (*n* = 1235 vs. 1036, *p* = .031). There was no difference in the detection rate of EGCs/HGINs between the two techniques (47/1828 vs. 36/1828, *p* = .181). However, within the neoplastic patients, LCI showed a greater capacity to diagnose differentiated EGCs/HGINs (40/57 vs. 27/57, *p* = .013). The analysis based on morphological type revealed that LCI was effective than WLI for type 0-IIc (31 and 24 lesions, respectively) and 0-IIa (11 and 9 lesions, respectively), although these differences were not significant. Among the EGCs detected, 20 were 0-IIa, 8 were 0-IIb and 55 were 0-IIc by using LCI and WLI. There was no significant difference in morphology between lesions in the WLI and LCI groups. Additionally, LCI greatly shortened the examination time compared with WLI (257 ± 99 s vs. 274 ± 94 s, *p* = .019).

**Table 3.** Endoscopic findings and examination time among LCI and WLI.

	LCI	WLI	*p* Value
Suspicious lesion	1235	1036	.031
EGC/HGINs	47/1828^a^	36/1828^a^	.181
Differentiated EGC/HGINs	40/57^b^	27/57^b^	.013
Macroscopic type			
0-IIa	11	9	.866
0-IIb	5	3	.724
0-IIc	31	24	.946
Examination time(s)	257 ± 99	274 ± 94	.019

^a^The enrolled patients.

^b^The neoplastic patients.

As shown in Supplementary Table 1, a total of 26 lesions were detected in the EGD examinations by both LCI and WLI; 23 (88.5%) of these lesions were classified as differentiated EGCs/HGINs and 3 (11.5%) were classified as undifferentiated EGCs. The remaining 31 out of 57 lesions were observed by only 1 of the 2 techniques. More lesions were detected by LCI alone (21 lesions) than by WLI alone (10 lesions) (*p* = .0078); the same was observed for differentiated EGCs/HGINs specifically, but the difference was not significant (*p* = .096).

The size and location of the EGCs/HGINs identified by LCI and WLI are shown in Supplementary Table 2. More EGCs/HGINs with diameters of ≤5 mm (*n* = 12 versus 6) and 6–10 mm (*n* = 8 versus 4) were detected by LCI than by WLI. Regarding location, a total of 12 lesions were detected by LCI in the lower 1/3 of the gastric lumen, which was the most common location; however, only two lesions were detected in that region by WLI alone. Four EGCs/HGINs detected by LCI alone were located in the upper third, and five were located in the middle third; the numbers detected by WLI alone in these regions were not significantly different.

As indicated in Supplementary Table 3, the detection rate of suspicious lesions across the 12 centres matched the theoretical prediction. The proportions of patients with single EGCs/HGINs, no suspicious lesions (*n* = 0) and multiple suspicious lesions (*n* ≥ 2) were relatively homogeneous.

## Discussion

In this prospective, multicentre RCT, LCI consistently showed better diagnostic performance than WLI in terms of accuracy, sensitivity and specificity (78.8%, 89.5% and 78.3%, respectively), indicating that LCI has superior accuracy and efficacy to WLI in the diagnosis of EGC. These results suggest that LCI may be useful in routine endoscopy for identifying EGC because it can reveal different colours in the gastric mucosa, which may influence the precision of diagnosis of malignant lesions.

As IEE screening for gastrointestinal malignant lesions has progressed, LCI has been demonstrated to be a brighter IEE technique than WLI and thus more useful in screening for lesions because it provides improved visualization of changes in redness, allowing easy recognition and detection of EGCs (18–20). Recently, two clinical randomized studies have reported the efficacy of LCI in detecting EGC. The LCI-FIND trial indicated that the detection rate of neoplastic lesions in the upper GI tract was 1.67 times higher by LCI than with WLI in high-risk patients examined by experienced endoscopists (21). In this trial, they also demonstrated that LCI is useful in identifying neoplastic lesions when used in ultraslim endoscopy and should be further promoted it in screening and surveillance tests (22). In another RCT from China, the detection rate of gastric neoplastic lesions was 8.01% in the LCI + WLI group, which was higher than that in the WLI-alone group in a high-risk population of gastric cancer patients (23).

Although our research and the previous trials all studied the detection of EGC by LCI, our research differs in some ways. First, the LCI-FIND trial included patients with known previous or current cancers of the GI tract (pharynx, oesophagus, stomach and large intestine) and the RCT in China included a high-risk population. In our study, the observations were performed on an average population that may be more representative of routine clinical practice. Second, these previous studies have used LCI alone, and because there was no diagnostic diagram for LCI to detect EGC, the endoscopists mainly relied on subjective observation. However, in China, because of the different experience levels of endoscopists, more careful training may improve the ability to identify EGC, especially for the new IEE technique of LCI. Third, in our study, the LCI diagnostic diagram may allow us to use LCI more efficiently to detect EGC (13). Thus, we demonstrated that LCI could enhance colour discrimination and improve the ability to discriminate between gastric neoplasia and non-neoplastic conditions. According to our previous study, the intraobserver and interobserver reproducibility tests for the LCI diagnostic diagram showed that the criteria for distal gastric diseases had good reliability for both experienced and inexperienced endoscopists (13). The training session and the increased colour difference between the lesion and surrounding mucosa might improve the visibility even for nonexpert endoscopists.

Until now, the endoscopic features of EGC in LCI mode were not well characterized, and the diagnostic efficiency based on the above criteria was also not detailed. In our study, we defined suspicious lesions and EGC under LCI to avoid misdiagnosis and further clarify the endoscopic characterization for precise diagnosis. In previous studies, EGCs appeared orange–red on LCI, and the surrounding mucosa was purple; these findings concur with those of our study. As we described in our study, the diagnosis of EGC by LCI was defined by the presence of a well-demarcated border and a yellow–red colour in the suspicious lesion. Then, according to the presence of purple in the peripheral mucosa, the lesion was categorized as differentiated or undifferentiated in type. Compared with other diagnostic methods, the unique colour signature of LCI made it easy to implement and improved the endoscopists’ diagnostic ability. Therefore, in detecting EGCs, LCI achieved approximately 10% higher accuracy than WLI, and the sensitivity was improved by more than 30%. Moreover, the examination time was significantly shortened, indicating that LCI can diagnose neoplastic lesions not only more precisely but also more rapidly than WLI in routine clinical practice.

The endoscopic findings were evaluated in greater detail using LCI, emphasizing the slight colour differences between the EGC and surrounding mucosa, which led to the detection of more lesions. A previous RCT considering the morphology of the lesions showed that, for both the 0-IIa and 0-IIc types, the LCI + WLI group had more neoplastic cases than the WLI group, as in our study. Additionally, published studies indicate that 0-IIc lesions are more readily detected by LCI than by WLI, and our study supports a potential role for LCI in improving the detection of 0-IIc lesions. Based on our investigations on the lower digestive tract, LCI is more effective than WLI at detecting flat lesions and lesions measuring <5 mm in diameter (10) and can observe the borders of laterally spreading tumours more clearly (24)^.^ These results indicate that LCI has an advantage in determining the border of the lesions. Furthermore, the results of published studies indicate that lesions with diameters ≤5 mm are more commonly detected by LCI than by WLI (10,21). Our study supports a potential role for LCI in improving the detection of small lesions due to its high sensitivity. One of the areas in which WLI is limited is the detection of small, depressed cancers or flat cancers, as their colour is similar to the surrounding reddish, erosive changes that occur in chronic gastritis. In our study, we detected 12 EGC/HGIN lesions measuring ≤5 mm by using LCI but only 6 such lesions by using WLI.

Large cross-sectional populations were enrolled at 12 hospitals, which examine populations different from the general population and from the high-risk populations reported in the literature. However, the participating centres were classified into two levels based on the number of people enrolled. Three measures, namely, the detection rates of EGCs/HGINs, no suspicious lesion (*n* = 0) and multiple suspicious lesions (*n* ≥ 2) (see Supplementary Table 3), were relatively homogeneous in all the hospitals. Furthermore, because the endoscopists with different experience levels were trained before the study and shown representative LCI images of EGCs, the endoscopists may have achieved good reliability and interobserver reproducibility in their measurements.

This study has some limitations. First, the prevalence of EGC was different at each institution, and the sample size may not be sufficient for evaluating all these outcomes. This may have resulted in the lack of difference in detection rates by the two methods. Accumulating more cases will be necessary to demonstrate the superiority of LCI in detecting EGC. Second, this study had 6 patients with undifferentiated EGC detected by WLI; previous literature has reported that undifferentiated EGC mainly presents endoscopically as discolouration that does not play to the advantages of LCI. Further studies are needed to demonstrate the LCI endoscopic features of undifferentiated EGC. Third, since magnifying endoscopy has been broadly used in the qualitative diagnosis of EGC and has shown higher diagnostic accuracy than WLI (3,4), our study did not use it, mainly because our main aim in the present study was to compare the difference in diagnostic accuracy between LCI and WLI with no magnification. One reason is that acquiring high-quality magnified images during endoscopy requires very steady movement of the endoscope, which can be particularly challenging for novice endoscopists and causes inevitable time costs; the other reason is that rapid screening is more suitable for China and is rarely influenced by the experience of endoscopists. In future studies, we will consider combining magnifying endoscopy and LCI to confirm whether their joint use further improves the sensitivity and specificity of EGC detection.

## Conclusion

In conclusion, this prospective, multicentre RCT revealed that LCI achieves better accuracy and a shorter examination time than WLI in diagnosing EGC; screening endoscopy of the stomach should be performed using LCI for routine clinical observation.

## Supplementary Material

Supplemental Material

## Data Availability

Due to patient privacy, all the data sets generated or analysed in this study are available from the corresponding author upon reasonable request (Yan Liu, 13911798288@163.com).
