# Supplementary material for: Diagnostic accuracy of linked colour imaging versus white light imaging for early gastric cancers: a prospective, multicentre, randomized controlled trial study
Source: Ann Med. 2022 Nov 21;54(1):3306–14. doi: 10.1080/07853890.2022.2147991 (PMC9704855; doi:10.1080/07853890.2022.2147991)
Supplement: Supplemental Material [file IANN_A_2147991_SM7845.docx]

Supplementary table 1. Per-lesion analysis of EGC detection among LCI and WLI

|  | **Detected LCI** | **Not detected LCI** | **P value** |
| --- | --- | --- | --- |
| Cancer/HGINs |  |  |  |
| Detected WL | 26 | 10 | 0.0078 |
| Not detected WL | 21 | 0 |  |
| Differentiated EGC/HGINs |  |  |  |
| Detected WL | 23 | 4 | 0.0960 |
| Not detected WL | 17 | 0 |  |

Supplementary table 2. Characteristics of EGC/HGINs using both LCI and WLI, LCI alone, or WLI alone.

| **Characteristics** | **Detected by LCI + WLI (n=26)** | **Detected by LCI only (n=21)** | **Detected by WLI only (n=10)** |
| --- | --- | --- | --- |
| **Size of lesion, mm** |  |  |  |
| ≤ 5mm | 10 | 12 | 6 |
| 6-10mm | 14 | 8 | 4 |
| >10mm | 2 | 1 | 0 |
| **Location, n** |  |  |  |
| **Upper third** | **8** | **4** | **5** |
| Anterior wall | 0 | 0 | 1 |
| Posterior wall | 3 | 2 | 1 |
| Lesser curvature | 4 | 1 | 0 |
| Greater curvature | 1 | 1 | 3 |
| **Middle third** | **6** | **5** | **3** |
| Anterior wall | 1 | 2 | 0 |
| Posterior wall | 2 | 0 | 2 |
| Lesser curvature | 1 | 2 | 0 |
| Greater curvature | 2 | 1 | 1 |
| **Lower third** | **12** | **12** | **2** |
| Anterior wall | 1 | 3 | 0 |
| Posterior wall | 3 | 2 | 0 |
| Lesser curvature | 4 | 4 | 2 |
| Greater curvature | 4 | 3 | 0 |

Supplementary table 3. Center-Specific Numbers of Examinations.

| **Center** | **Endoscopist** | **Patients** | **EGC**  **/HGIN** | **Suspicious lesion(n**  **=0)** | **Multiple suspicious lesion(n**  **≥2)** |
| --- | --- | --- | --- | --- | --- |
| **1** | **2** | **25** | **3（12%）** | **5（20%）** | **4（16%）** |
| **2** | **2** | **32** | **1（3%）** | **0（0%）** | **6（19%）** |
| **3** | **2** | **36** | **2（6%）** | **4（11%）** | **9（25%）** |
| **4** | **2** | **42** | **2（5%）** | **3（7%）** | **0（0%）** |
| **5** | **2** | **57** | **2（4%）** | **3（5%）** | **12（21%）** |
| **6** | **3** | **86** | **0（0%）** | **15（17%）** | **16（19%）** |
| **7** | **2** | **177** | **9（5%）** | **11（6%）** | **34（19%）** |
| **8** | **2** | **212** | **9（4%）** | **19（9%）** | **42（20%）** |
| **9** | **3** | **247** | **5（2%）** | **12（5%）** | **66（27%）** |
| **10** | **3** | **266** | **8（3%）** | **20（8%）** | **57（21%）** |
| **11** | **2** | **282** | **11（4%）** | **20（7%）** | **62（22%）** |
| **12** | **3** | **362** | **5（1%）** | **22（6%）** | **71（20%）** |
